# Transvenous lead extraction using the TightRail mechanical rotating dilator sheath for Asian patients

**DOI:** 10.1038/s41598-021-99901-w

**Published:** 2022-01-17

**Authors:** Ji-Hoon Choi, Seung-Jung Park, Hye Ree Kim, Hee-Jin Kwon, Kyoung-Min Park, Young Keun On, June Soo Kim, Ju Youn Kim, Won Young Jung

**Affiliations:** Division of Cardiology, Department of Medicine, Heart Vascular and Stroke Institute, Samsung Medical Center, Sungkyunkwan University School of Medicine, 81 Irwon-ro, Gangnam-gu, Seoul, 06351 Republic of Korea

**Keywords:** Cardiac device therapy, Arrhythmias

## Abstract

The need for transvenous lead extraction (TLE) is increasing worldwide including in Asia–Pacific regions. However, supporting evidence for TightRail, a relatively new rotating mechanical dilator sheath, is still lacking in Asian patients. The efficacy and safety of TLE using TightRail performed between March 2018 and June 2021 were evaluated in 86 consecutive patients with 131 leads. The mean lead age was 11.7 ± 7.3 (range, 1.0–41.4) years. Clinical and complete procedural success using TightRail were achieved in 93.0% and 89.5% of 86 patients, respectively, with 6 min of median fluoroscopic time and 9.3% of major complication rate: death (1.2%), cardiac tamponade (3.5%), severe tricuspid regurgitation (3.5%), and stroke (1.2%). However, in 46 patients with longest lead age ≤ 10 years, clinical/complete success and major cardiac complication rates turned out better as 97.8%, 95.7%, and 2.2%, respectively. Additionally, when patients were divided into 3 groups: the first 28, second 29, and the last 29 patients, there was a clear trend toward better efficacy and safety outcomes with more experience with TightRail (P_trend_ < 0.05). Longest lead age > 10 years was closely associated with TLE-related major cardiac complication (P = 0.046) with 85.7% sensitivity, 57.0% specificity, 15.0% positive predictive value, and 97.8% negative predictive values. In conclusion, TLE using TightRail may be effectively and safely performed by experienced operators for Asian patients with the longest lead age ≤ 10 years. However, as TightRail is a potentially aggressive tool, special attention should be paid to patients with longer lead dwelling times (e.g., > 10 years).

## Introduction

Recently, the number of cardiac implantable electronic device (CIED) implantations has dramatically increased worldwide, including in Asia–Pacific regions^1–3^. In parallel with the increasing number of CIED implantations, the need for lead extraction is also increasing significantly due to the rising CIED-related infections, lead malfunctions, and CIED upgrades^4–6^. Specialized tools and techniques are required to extract leads implanted over one year because of the formation of extensive fibrotic adhesions between the leads and various cardiovascular structures^7^. Lead extraction technologies have made significant progress over the past 40 years, and now transvenous lead extraction (TLE) is the preferred method for lead revision and system upgrade^6^. A recent study on a large prospective registry of TLEs in Europe showed that the current practice of TLE is associated with a high success rate with a low complication rate^8^. However, life-threatening or non-lethal complications such as cardiac tear with tamponade, stroke, and tricuspid valve (TV) injury do occur during TLEs, even in those performed by experienced operators^4,9,10^. Thus, knowledge of the efficacy and safety of various specialized tools and techniques for TLE is essential, from the simple telescoping sheaths to several powered sheaths. Currently, most of the efficacy and safety data are derived from patients in Western countries.

**Figure 1:**
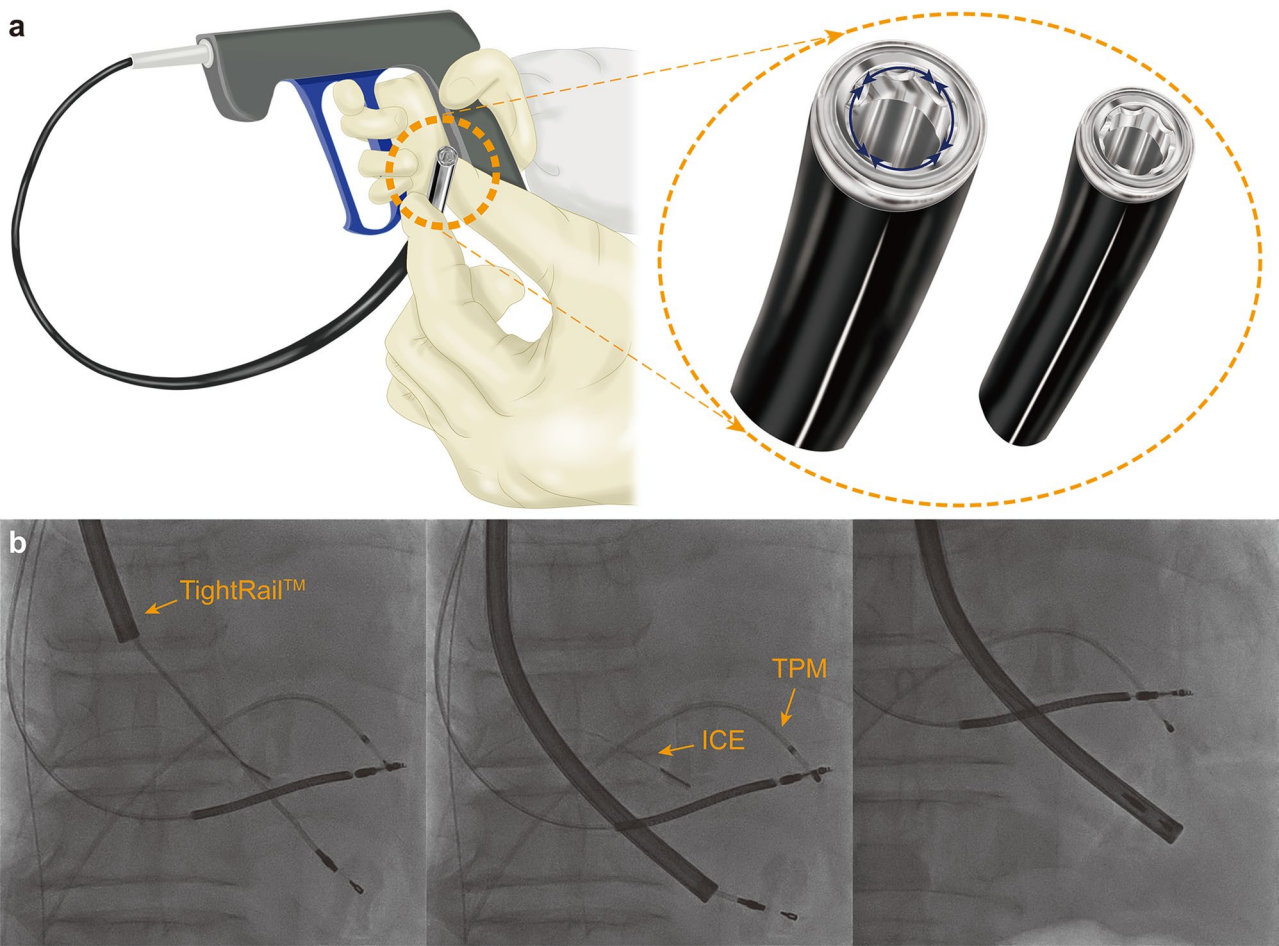
TightRail mechanical dilator sheath with bidirectionally rotating blade (**a**) and serial fluoroscopic image showing TLE procedure using TightRail sheath under ICE and temporary pacing wire backup (**b**). *ICE* intracardiac echocardiography probe, *TLE* transvenous lead extraction, *TPM* temporary pacemaker.

TightRail (Spectranetics Corp., Colorado Springs, CO, USA) is a relatively new mechanical dilator sheath with a bidirectional rotating blade shielded inside a flexible shaft (Fig. 1a). As this novel tool has been used less frequently in Asia–Pacific regions, supporting evidence for use of this instrument in Asian patients is still lacking. Therefore, we report our initial experience with the TightRail sheath in terms of both safety and efficacy, and we suggest lead characteristics that could predict periprocedural adverse events.

## Methods

### Study population

Since June 2014, clinical, electrocardiographic, echocardiographic, device- and procedure-related variables from consecutive patients undergoing TLE procedures at our institution have been prospectively collected and entered into a procedural database and electronic medical record. For the present retrospective study, all consecutive patients who underwent TLE using TightRail in our center from March 2018 to June 2021 were included. TLE was defined as either removal of lead(s) implanted for > 1 year or lead removal requiring specialized extraction equipment not used during implantation. Patients with a lead age of less than one year were excluded from the study. Patients in whom only simple traction was required to remove the leads without specialized extraction tools were also excluded. The indication for TLE was determined according to the 2017 Heart Rhythm Society (HRS) expert consensus statement on CIED lead management and extraction^11^. Lead malfunction was defined as a clinically significant alteration in lead impedance, sensing, or pacing threshold. The diagnosis of CIED-related endocarditis was based on the 2019 International CIED Infection Criteria^12^. Patient with local signs of pocket infection but no vegetation was classified as isolated pocket infection^12^. Informed consent was obtained from all patients before study enrollment. This study complied with the Declaration of Helsinki, and the research protocol was approved by the local institutional ethics board (Samsung Medical Center Institutional Review Board).

### Lead extraction procedure

TLE was performed under deep sedation using midazolam and propofol in the electrophysiology laboratory with continuous monitoring of arterial blood pressure and oxygen saturation. Real-time intracardiac echocardiography was routinely performed during the entire procedure using the AcuNav Ultrasound Catheter (Biosense Webster Inc., Diamond Bar, CA, USA) to visualize cardiac structure and to identify pericardial effusion immediately (Fig. 1b). In cases of pacemaker dependency, temporary transvenous pacing was established. The cardiac surgeon was on standby during all procedures to perform emergent operations. One experienced main operator and one or two assistants who specialized in device management conducted all procedures. All TLE procedures were performed through the implant sites, subclavian veins. After local anesthesia, the generator pocket was opened and careful dissection was performed to release the rolled leads. The lead-locking device (Lead Locking Device, Spectranetics Corp., Colorado Springs, CO, USA) was inserted into the distal lumen of the lead and then tied tightly with the lead using surgical suture. The dilator sheath was introduced over the lead to the venous entry site and aligned with the lead direction using fluoroscopic guidance. Triggering of the TightRail was carefully performed, observing the dilator sheath advancing little by little along the leads. In some cases, a simple telescoping sheath was used as an adjunct tool to overcome tight stenotic lesions between the first rib and clavicle. The dilator sheath was advanced until the lead tip was completely separated from the heart. After the TLE, a new CIED system was implanted at the same session in patients in need of device replacement. In cases of CIED-related infection, device re-implantation was postponed until the complete eradication of infectious microorganisms according to the current guideline^12^. All patients were transferred to the cardiac care unit for close monitoring for 1 or 2 days after the TLE procedure. Transthoracic echocardiographic (TTE) was performed before and after procedure to assess TLE-related cardiac complications using commercially available equipment (Vivid 9 or 7 GE Healthcare, Chicago, IL, USA).

### Definitions

The definitions of success and complications were based on the 2017 HRS expert consensus statement on CIED lead management and extraction^11^. Complete procedural success was defined as the removal of all targeted leads and material from the vascular space with the absence of any permanently disabling complication or procedure-related death. Clinical procedural success was defined as the removal of all targeted leads and lead material from the vascular space or retention of a small portion of the lead (< 4 cm) that does not negatively impact the outcome goals of the procedure in the absence of complications requiring surgical intervention. Major complications were defined as procedure-related death, life-threatening outcomes requiring immediate surgical intervention, or permanent injury of the body such as a cardiac tear, cardiac tamponade, new-onset severe tricuspid regurgitation (TR), and stroke. Major cardiac complications were defined as major complications except stroke for the present study. Non-major complications were defined as non-life-threatening outcomes requiring only medical treatment or minor procedural intervention. Pre- and post-TLE TR severity score was graded as trivial (or minimal), mild, moderate, or severe according to international guidelines^13^. A significant TR aggravation was defined as an increase of at least 1 grade with a post-extraction TR ≥ moderate whereas TR improvement as a decrease of at least 1 grade^4^. The procedure time was calculated from the time the dilator sheath was first mounted on the leads to the time the lead was completely removed from the body. Fluoroscopic time was total fluoroscopy exposure time accumulated during the TLE.

### Statistical analysis

Continuous variables are reported as mean ± standard deviation (SD) or median with inter-quartile range (IQR), and all categorical variables are reported as the number and percentage. To compare the two groups, Mann–Whitney test for continuous variables and the chi-square or Fisher’s exact test for categorical variables were used. To compare the three groups, analysis of variance test for continuous variables and the Kruskal–Wallis test for categorical variables were used. P for trend was calculated using Cochran-Armitage test. Receiver operating characteristic (ROC) curve analysis was performed to evaluate the optimal cut-off value of the longest lead age for predicting major cardiac complications. All statistical tests were two-tailed and a P value of < 0.05 was considered significant. All statistical analyses were performed using SPSS statistical software version 25 (SPSS Inc., Chicago, IL, USA).

## Results

### Baseline patient and lead characteristics

The TightRail mechanical dilator sheath was first used at our institution in March 2018. Since then, this device had been used for TLE of 131 leads from 86 patients by June 2021. The baseline characteristics of the study population are summarized in Table 1. The mean patient age was 66.3 ± 14.1 years (range 24–88 years), and 47 (54.7%) patients were male. The removed generators were permanent pacemakers in 69 (80.2%) patients, implantable cardioverter-defibrillators in 15 (17.4%) patients, and cardiac resynchronization therapy-defibrillator devices in two (2.3%) patients. The most common indication was lead malfunction in 51 (59.3%) patients, followed by infection (CIED-related endocarditis and/or isolated pocket infection) in 15 (17.4%) patients, and device upgrade in 15 (17.4%) patients (Table 1). Five additional patients underwent lead extraction due to radiation therapy, chronic pain, and phrenic nerve stimulation. The characteristics of extracted leads are provided in Table 2. Extracted leads were 73 (55.7%) right ventricular (RV) pacing leads, 40 (30.5%) atrial leads, 17 (13.0%) defibrillation leads, and one (0.8%) left ventricular lead. The mean lead age was 11.7 ± 7.3 years. In 40 patients (46.5%), the longest lead age was longer than 10 years, and 37 patients (43.0%) underwent extraction of two or more leads during the same session.

**Table 1:** Demographic and baseline characteristics.

Clinical variables	N = 86
Age, years	66.3 ± 14.1
Male, n (%)	47 (54.7)
Body mass index, kg/m^2^	24.5 ± 3.9
Hypertension, n (%)	48 (55.8)
Diabetes mellitus, n (%)	22 (25.6)
Coronary artery disease, n (%)	12 (14.0)
Chronic kidney disease n (%)	22 (25.6)
Left ventricular ejection fraction, %	52.7 ± 14.1
Left ventricular ejection fraction < 40%, n (%)	20 (23.3)
Antiplatelet agents, n (%)	26 (30.2)
Oral anticoagulant, n (%)	19 (22.1)
**Device-related variables**	
***Type of implanted generator***	
Pacemaker, n (%)	69 (80.2)
Single-chamber, n (%)	21 (24.4)
Dual-chamber, n (%)	48 (55.8)
ICD, n (%)	15 (17.4)
Single-chamber, n (%)	15 (17.4)
Dual-chamber, n (%)	0
CRT-D, n (%)	2 (2.3)
***Indication of lead removal***	
Lead malfunction, n (%)	51 (59.3)
Endocarditis, n (%)	8 (9.3)
Pocket infection, n (%)	7 (8.1)
Device upgrade, n (%)	15 (17.4)
Others, n (%)	5 (5.8)
Ventricular-pacing ≥ 95%, n (%)	45 (52.3)

*CRT-D* cardiac resynchronization therapy-defibrillator, *ICD* implantable cardioverter-defibrillator.

**Table 2:** Characteristics of extracted leads.

Total number of extracted leads	N = 131
**Type of extracted leads**	
Right atrial lead, n (%)	40 (30.5)
Right ventricular lead, n (%)	90 (68.7)
Pacing lead, n (%)	73 (55.7)
Single-coil ICD lead, n (%)	5 (3.8)
Dual-coil ICD lead, n (%)	12 (9.2)
Left ventricular pacing lead, n (%)	1 (0.8)
**Fixation type**	
Passive fixation, n (%)	79 (60.3)
Active fixation, n (%)	52 (39.7)
**Sheath diameter**	
11 Fr, n (%)	94 (71.8)
13 Fr, n (%)	32 (24.4)
Both, n (%)	5 (3.8)
**Age of extracted leads**	
Range, years	1.0–41.4
Mean with SD, years	11.7 ± 7.3
Median with IQR, years	9.5 (6.8–15.2)

Longest lead age per patient	N = 86
1 − 5 years, n (%)	9 (10.5)
5 − 10 years, n (%)	37 (43.0)
10 − 15 years, n (%)	18 (20.9)
15 − 20 years, n (%)	9 (10.5)
≥ 20 years, n (%)	13 (15.1)

Combined lead age per patient	N = 86
1–10 years, n (%)	32 (37.2)
10–20 years, n (%)	30 (34.9)
20–30 years, n (%)	8 (9.3)
30–40 years, n (%)	5 (5.8)
≥ 40 years, n (%)	11 (12.8)

Number of extracted leads per patient	N = 86
One lead extracted, n (%)	49 (57.0)
Two leads extracted, n (%)	30 (34.9)
Three leads extracted, n (%)	6 (7.0)
Four leads extracted, n (%)	1 (1.2)

*Fr* French, *ICD* implantable cardioverter-defibrillator, *IQR* interquartile range.

### Acute procedural and post-discharge outcomes

Overall clinical success was achieved in 80 (93.0%) patients with the median (IQR) procedural and fluoroscopic time of 10.0 (5.0–23.0) min and 6.0 (3.0–11.5) min, respectively. Complete procedural success rate using the TightRail system was 89.5% (77/86). For 20 (23.3%) patients, telescoping sheaths were also used to predilate the stenotic entry site between the first rib and clavicle. Other tools such as snares or laser sheaths were not used. Major complications were encountered in eight patients (9.3%). Detailed information on all cases with major complications is presented in Supplementary Table S1. There was one case of sudden death, which occurred in an 83-year-old female patient 2 hours post-extraction of 2 RV pacing leads with 21.0 and 16.8 years of lead ages, respectively. Delayed tamponade was suspected by bedside echocardiography as the cause of her sudden collapse. Cardiac tamponade occurred immediately after (n = 2) or during (n = 1) the TLE procedures in three additional patients. Emergent cardiac surgeries showed two cases of right atrial (RA) tear (a 15.1-year-old RV pacing lead with atrial sensing ring and a 23.6-year-old RA lead) and one case of RV tear (RV pacing lead with dwell time of 11.7 years). New-onset Severe TR was confirmed in three patients (RV pacing leads with lead ages of 21.6, 10.1, and 9.0 years, respectively) on post-procedural TTE. One patient underwent elective TV repair surgery whereas the remaining two patients showed no RV dysfunction on the follow-up TTE and remained asymptomatic with medical treatment alone. Embolic stroke occurred in a 57-year-old male patient with hypertrophic cardiomyopathy and atrial fibrillation 2 days after the extraction procedure. Anticoagulation had been discontinued for 2 days before the procedure. Emergent thrombolysis was performed, and he was discharged 30 days after lead extraction with mild dysarthria. Non-major complications were observed in five patients including pocket hematoma (n = 1), moderate-sized pericardial effusion (n = 1), and new-onset moderate TR (n = 3). The pocket hematoma and pericardial effusion resolved spontaneously without invasive treatments. Overall, significant TR aggravation was identified in 6 (7.0%) patients whereas TR improvement defined as a decrease of at least 1 grade was observed in 5 (5.8%) patients following TLE. Details on the changes in the TV function before and after the TLE were presented in Supplementary Table S2 and S3. There was one incomplete extraction case performed for lead malfunction in a 56-year-old male, where atrial lead tip (2 cm, lead age of 22.5 years) with passive fixation remained in the RA appendage.

After discharge, all patients except the one in-hospital death case were followed up for 301 (93–580) days without further adverse events including CIED-related infection, heart failure admission, stroke, or death.

### Risk factors for major cardiac complications

In total, seven cases of major cardiac complications developed during or immediately after the TLE, which included death (n = 1), cardiac tamponade (n = 3), and new-onset severe TR (n = 3). Patients with major cardiac complication exhibited significantly greater longest (15.1 [10.1–21.6] vs. 9.0 [6.8–13.9] years; P = 0.029) and combined lead ages (20.3 [15.1–43.1] vs. 11.2 [7.4–20.1] years; P = 0.020) than those without (Table 3). In particular, the longest lead ages > 10 years (85.7% vs. 43.0%, P = 0.046) and passive fixation (100.0% vs. 58.2%, P = 0.040) were more frequently found in patients with major cardiac complications than in those without. Procedure time and post-extraction hospital stay were also significantly longer in patients with major cardiac complications than in those without. However, other variables were not significantly different between the two groups such as age, sex, comorbidities, use of medications, left ventricular systolic function, ventricular pacing dependency, lead types (atrial, ventricular, pacing, or defibrillation), a number of extracted leads, and diameter of TightRail sheath used (Table 3).

**Table 3:** Comparison between patients with and without major cardiac complications.

	Major cardiac complication (n = 7)	No major cardiac complication (n = 79)	P-value
Age, years	74.0 (72.0–84.7)	68.0 (57.0–75.0)	0.053
Male, n (%)	2 (28.6)	45 (57.0)	0.237
Body mass index, kg/m^2^	24.3 (23.0–27.6)	23.7 (22.4–26.3)	0.653
Hypertension, n (%)	5 (71.4)	43 (54.4)	0.457
Diabetes mellitus, n (%)	3 (42.9)	19 (24.1)	0.365
Chronic kidney disease, n (%)	3 (42.9)	20 (25.3)	0.378
Coronary artery disease, n (%)	0	12 (15.2)	0.586
Anti-platelet agent, n (%)	2 (28.6)	24 (30.4)	1.000
Anticoagulation, n (%)	2 (28.6)	17 (21.5)	0.647
Left ventricular ejection fraction, %	60.0 (60.0–64.0)	57.0 (38.6–62.0)	0.197
Ventricular-pacing ≥ 95%, n (%)	5 (71.4)	40 (50.6)	0.437
PPM-dual chamber, n (%)	5 (71.4)	43 (54.4)	0.457
PPM-single chamber, n (%)	2 (28.6)	19 (24.1)	1.000
ICD-single chamber, n (%)	0	15 (19.0)	0.346
Number of extracted leads, n (%)	2 (1–2)	1 (1–2)	0.122
Longest lead age, years	15.1 (10.1–21.6)	9.0 (6.8–13.9)	0.029
Longest lead age ≥ 10 years, n (%)	6 (85.7)	34 (43.0)	0.046
Combined lead age, years	20.3 (15.1–43.1)	11.2 (7.4–20.1)	0.020
RA-pacing lead, n (%)	3 (42.9)	33 (41.8)	1.000
RV-pacing lead, n (%)	7 (100.0)	57 (72.2)	0.183
Single coil ICD lead, n (%)	0	5 (6.3)	1.000
Dual coil ICD lead, n (%)	0	12 (15.2)	0.586
Passive fixation, n (%)	7 (100.0)	46 (58.2)	0.040
Use of 13-Fr sheath, n (%)	1 (14.3)	26 (32.9)	0.425
Procedure time, mins	25.0 (24.0–33.0)	8.0 (4.7–21.0)	0.037
Post-extraction hospital stay, days	24 (19–30)	3 (3–5)	< 0.001

Abbreviations: Fr, French; ICD, implantable cardioverter-defibrillator; PPM, permanent pacemaker; RA, right atrial; RV, right ventricular.

**Figure 2:**
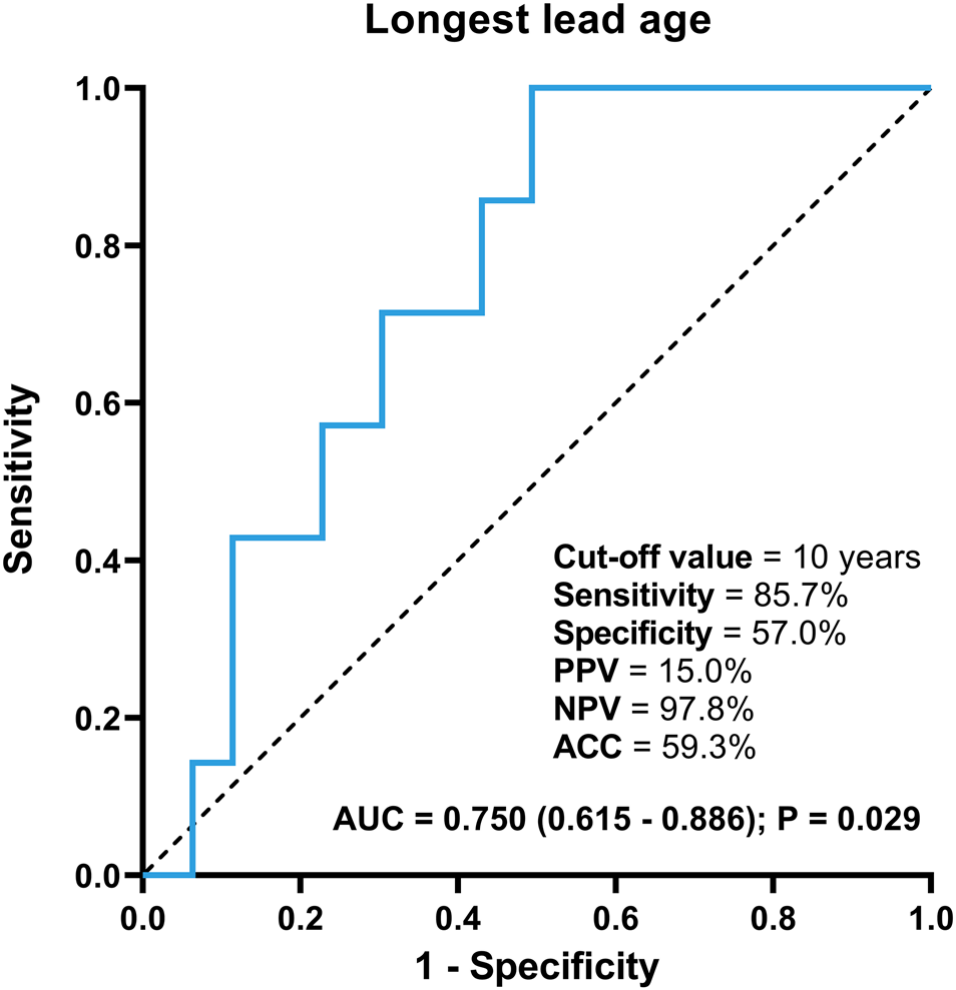
Receiver operating characteristic curve for the longest lead age in predicting clinically significant pericardial effusion. *ACC* accuracy, *AUC* area under the curve, *NPV* negative predictive value, *PPV* positive predictive value**.**

The ROC analysis showed that the optimal cut-off value in the longest lead age for predicting major cardiac complication was determined around 10 years (Fig. 2) with 85.7% sensitivity, 57.0% specificity, and 97.8% negative and 15.0% positive predictive values. The area under the ROC curve was 0.750 (95% CI: 0.615–0.886; P = 0.029).

### Post-hoc analysis on the efficacy and safety outcomes

To see the effect of the lead age on the overall outcomes, we performed post-hoc tests, dividing the patients into two subgroups: patients with the longest lead age ≤ 10 years (n = 46, Below10 group) and those with longest lead age > 10 years (n = 40, Above10 group). The longest lead age was significantly longer in the Above10 than in the Below10 groups (17.4 ± 7.0 vs. 6.7 ± 2.3 years, P < 0.001). The safety and efficacy outcomes were likely to be better in the Below10 compared to those in the Above10 group as presented in Table 4. All leads were successfully extracted in the Below10 group without lead remnants or major complications except one case of stroke and another case of new-onset severe TR requiring no surgical treatment.

**Table 4:** Procedural outcome according to longest lead age.

	Above10^a^ (n = 40)	Below10^a^ (n = 46)	P-value
Longest lead age, years (mean)	17.41 ± 6.95	6.74 ± 2.25	
Longest lead age, years (median)	15.94 (11.55–21.69)	6.99 (5.38–8.50)	
Complete success, n (%)	33 (82.5)	44 (95.7)	0.075
Clinical success, n (%)	35 (87.5)	45 (97.8)	0.092
Major complications, n (%)	6 (15.0)	2 (4.3)	0.138
Major cardiac complications, n (%)	6 (15.0)	1 (2.2)	0.046

^a^Indicates patients with longest lead age > 10 years and ≤ 10 years, respectively.

**Figure 3:**
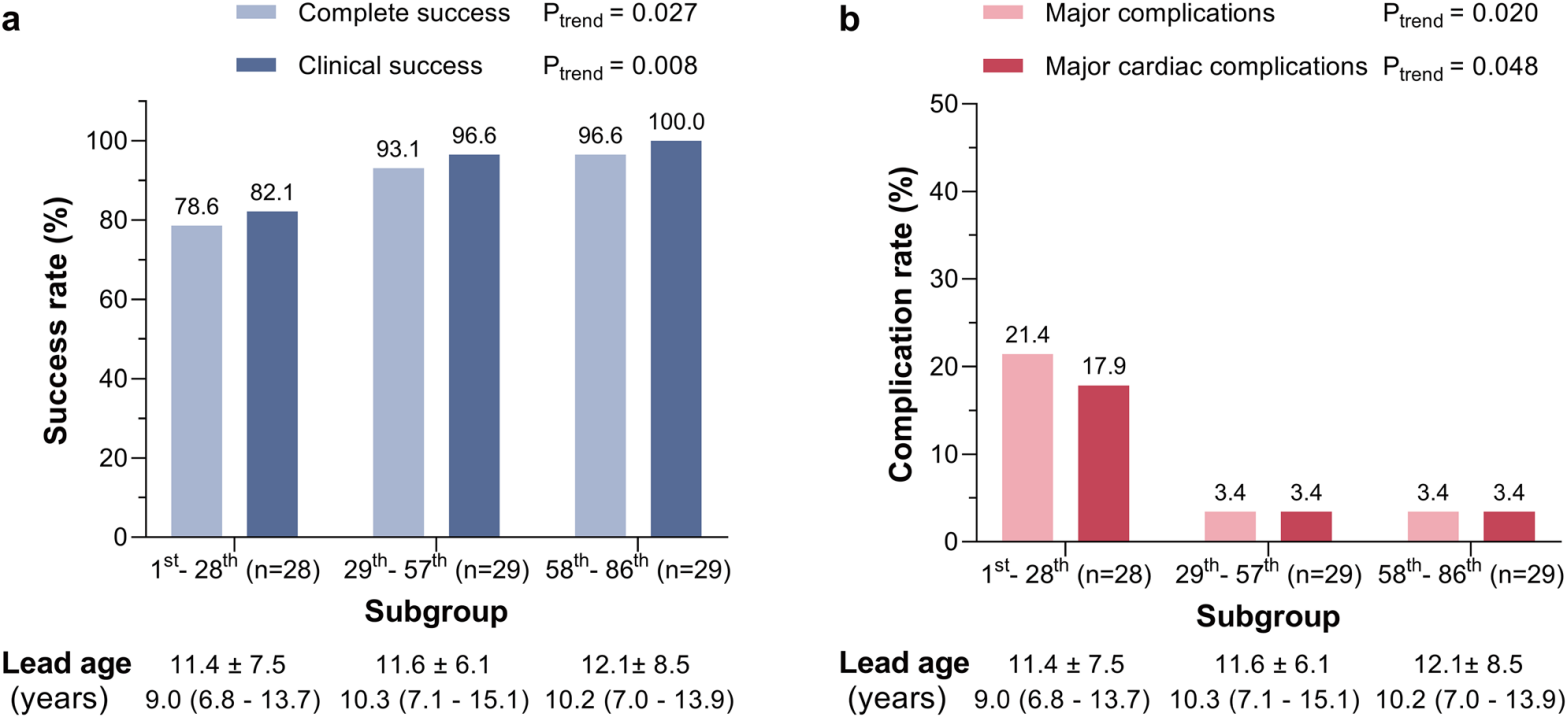
Change in outcomes according to experience with TightRail system**.** Patients were divided into three subgroups according to the accumulating experience with extraction procedure using TightRail system. A significant trend toward better efficacy (**a**) and safety (**b**) outcomes with more experience was observed although lead age was not significantly different among the three subgroups.

Additionally, the effect of learning curve with utilizing the TightRail system was also evaluated, dividing patients into three subgroups: the first 28, second 29, and the last 29 patients according to the experience with TLE procedures using TightRail. There was a significant trend toward better efficacy and safety outcomes with more experience although lead ages were not significantly different among the three subgroups (Fig. 3). Overall outcomes began to improve greatly from the second thirds of procedures. In the last 29-patient group, clinical and complete success rates were 96.6% and 100%, respectively, with major complication rate of 3.4%.

## Discussion

### Main findings

Our main findings were: (1) clinical and complete procedural success were achieved using TightRail in 80 (93.0%) and 77 (89.5%) of 86 patients, respectively, with mean lead age of 11.7 ± 7.3 years; (2) TLE-related major and major cardiac complications occurred in eight (9.3%) and seven (8.1%) patients, respectively; (3) the longest lead ages was significantly longer in patients with major cardiac complications than in those without, and the optimal cut-off value in predicting TLE-related major cardiac complication was also determined to be 10 years by ROC analysis; and (4) there was a clear trend toward better efficacy and safety outcomes with more experience with TightRail or with patients with the longest lead age ≤ 10 years.

### Efficacy and safety of TLE using TightRail

To the best of our knowledge, this is the first study to evaluate the efficacy and safety of the TightRail in the Asia–Pacific region. Previously, performance of this device was primarily analyzed in three Western countries. Aytemir et al. reported single-center experience in 23 patients with median lead age of 6 years^14^, another single-center study by Cay et al. was carried out in 40 patients with a mean lead age of 5.7 years^15^, and bicentric data by Mazzone et al. were obtained in 26 patients with mean lead age of 8.3 years^16^. Compared to the outcomes in these three studies, a relatively higher complication rate was observed in our data. Only one death case was reported in one of the previous studies due to superior vena cava/right atrium junction tear^15^. This discrepancy was probably associated with longer lead age (11.7 ± 7.3 years) in our study population compared to that in previous data. Post-hoc subgroup analyses revealed that most of the major complications occurred in patients with longest lead age > 10 years (Table 4). In contrast, in the patients with longest lead age ≤ 10 years, clinical and complete success rates were achieved in 97.8% and 95.7% of patients and the rate of major cardiac complications dropped to 2.2%.

In addition, learning curve with this new extraction tool may also affect our overall outcomes. Figure 3 demonstrated that a clear and significant trend toward better efficacy and safety outcomes with more experience with TLE using TightRail although the mean lead ages were not significantly different among the three subgroups. From the second thirds of procedures, complication rates began to decrease greatly, elevating clinical and complete success rates. In the last 29-patient subgroup which was most recently treated with more experience, our clinical and complete success rates (100% and 96.6%, respectively) became eventually comparable to the clinical success rates in previous studies above-mentioned (100%, 97.5%, and 100%)^14–16^. Moreover, our fluoroscopic time (median, 6.0 min) was not significantly different from the previous value (median, 5 min) reported by Aytemir et al.^14^. All of our patients with major complications except for the one death case, were successfully managed by medical and/or surgical treatments, eventually discharged, and were followed up uneventfully for 301 (93–580) days.

### Risk factors for major cardiac complications

Cardiac tamponade due to cardiac avulsion or tear is one of the most devastating complications encountered in patients undergoing TLE. A large retrospective study that included 91,890 TLEs in the United States, showed that the incidence of cardiac tamponade was 0.8%^17^. A recent European lead extraction registry that included 3510 TLEs reported that the incidence of cardiac tamponade due to cardiac avulsion or tear was 0.9%^18^. According to this study, 20% of patients with cardiac tamponade died despite pericardiocentesis or surgical repair. Therefore, knowledge of the risk factors for cardiac tamponade is of great importance. In our study, all of patients with cardiac tamponade (n = 4, 4.7%) had leads implanted for more than 10 years. The longest lead age over 10 years was a useful predictor for the occurrence of major cardiac complications including tamponade and new-onset severe TR. Interestingly, similar findings have recently been published in European Lead Extraction Controlled Registry data. These data showed that patients with lead age longer than 10 years are 5.1 times more likely to develop cardiac tamponade due to cardiac avulsion or tear^18^. Therefore, particular care should be taken while performing TLE of leads with longer dwell times (i.e., > 10 years).

TR aggravation is not uncommon after TLE. Although the incidence after TLE can vary depending on the extraction method, definition, and surveillance modality, it has been reported to be about 5–15%^19–22^. In a retrospective study including 208 consecutive patients undergoing TLE using mostly laser sheaths, lead implant duration was identified as an independent predictor of TLE-related acute TR aggravation^4^. In our study as well, a significant TR aggravation was observed in six (7.0%) patients with longest lead age greater than or around 10 years (21.6, 11.6, 10.3, 10.1, 9.4, and 9.0 years).

### Limitations

This study has the limitations inherent to a retrospective analysis with small sample size, which may bias the results and preclude any definite conclusion. So, our results need to be interpreted with caution. However, all TLE procedures were performed by one experienced cardiologist using the same treatment protocol. No comparison was made between TightRail and other powered sheaths in the present study. Therefore, multicenter prospective studies with a larger sample size need to be conducted to validate and expand upon our findings and to determine the risk factors for TightRail-associated significant complications.

## Conclusions

In our experiences, TLE using TightRail may be performed by experienced operators with acceptable efficacy and safety for Asian patients with the longest lead age ≤ 10 years just as for patients in Western countries. However, as TightRail is a potentially aggressive tool, special attention should be paid to patients with longer lead dwelling times (e.g., > 10 years), considering the high incidence of cardiac complications.

## Supplementary Information

Supplementary Tables.

## Data Availability

The data that support the findings of this study are available on request from the corresponding author. The data are not publicly available due to privacy or ethical restrictions.
