# Supplementary material for: Transvenous lead extraction using the TightRail mechanical rotating dilator sheath for Asian patients
Source: Sci Rep. 2022 Jan 17;11:22251. doi: 10.1038/s41598-021-99901-w (PMC8764071; doi:10.1038/s41598-021-99901-w)
Supplement: Supplementary file 1 — Supplementary Tables. [file 41598_2021_99901_MOESM1_ESM.docx]

**Supplementary Table S1.** Detailed information on patients with major complications.

| **No.** | **Sex** | **Age** | **LVEF**  **(**%**)** | **Extracted leads**  **(lead age,** years**)** | **Fixation** | **Lead location** | **Complications** | **Timing of**  **complications** | **Outcomes and comments** |
| --- | --- | --- | --- | --- | --- | --- | --- | --- | --- |
| 1 | F | 83 | 60 | RV-pacing lead (21.0) | Passive | RV apex | Sudden Death (delayed tamponade  suspected) | 2 hours  post-TLE | In-hospital death |
|  |  |  |  | RV-pacing lead (16.8) | Passive | RV apex |  |  |  |
| 2 | F | 85 | 60 | VDD lead  (15.1) | Passive | RV apex | Cardiac tamponade due to RA tear | During VDD lead extraction | RA wall reconstruction, Surgical removal of the VDD lead, Discharge alive 27 days post-TLE |
| 3 | F | 74 | 60 | RV-pacing lead (11.7) | Passive | RV apex | Cardiac tamponade due to RV tear | Right after RV lead extraction | Primary closure of RV, Discharge alive 30 days post-TLE |
| 4 | M | 58 | 52 | RA-pacing lead (12.6) | Passive | RAA | Cardiac tamponade due to RAA tear | Right after extraction of older RA lead | Primary closure of RAA, Successful TLE of the remaining RV lead 4 days after emergency operation, Discharge alive 20 days post-TLE |
|  |  |  |  | RA-pacing lead (23.6) | Passive | RAA |  |  |  |
|  |  |  |  | RV-pacing lead (23.6) | Passive | RV apex |  |  |  |
| 5 | F | 85 | 61 | RV-pacing lead  lead (10.1) | Passive | RV apex | Severe TR due to chordae rupture | Right after RV lead extraction | Discharged 19 days post-TLE, No RV dysfunction on follow-up echo, Remained asymptomatic with medical treatment alone |
|  |  |  |  | RA-pacing lead (10.1) | Passive | RAA |  |  |  |
| 6 | M | 72 | 64 | RA-pacing lead  (9.0) | Passive | RAA | Severe TR due to chordae rupture | Right after RV lead extraction | Discharged 15 days post-TLE, No RV dysfunction on follow-up echo, Remained asymptomatic with medical treatment alone |
|  |  |  |  | RV-pacing lead  (9.0) | Passive | RV apex |  |  |  |
| 7 | F | 72 | 64 | RA-pacing lead  (21.6) | Passive | RAA | Severe TR due to chordae rupture | Right after RV lead extraction | Elective tricuspid valve repair, Discharged alive 33 days  post-TLE |
|  |  |  |  | RV-pacing lead  (21.6) | Passive | RV apex |  |  |  |
| 8 | M | 57 | 57 | Dual-coil ICD lead (8.1) | Active | RV apex | Embolic stroke | 2 days post-TLE | Emergent thrombolysis, Discharged alive 30 days post-TLE with a mild dysarthria |

Abbreviations: ICD, implantable cardioverter-defibrillator; PPM, permanent pacemaker; RA, right atrium; RAA, RA appendage; RV, right ventricle; VDD, ventricular pacing dual chamber sensing dual chamber function lead; TLE, transvenous lead extraction; TR, tricuspid regurgitation.

**Supplementary Table S2.** Details on changes in tricuspid valve function.

| **TR Severity and Score** | **Pre-TLE** | **Post-TLE** | **P-value*** |
| --- | --- | --- | --- |
| **TR severity** |  |  |  |
| Trivial or minimal=1, n (%) | 41 (47.7) | 40 (46.5) | 0.716 |
| Mild=2, n (%) | 34 (39.5) | 30 (34.9) |  |
| Moderate=3, n (%) | 9 (10.5) | 12 (14.0) |  |
| Severe=4, n (%) | 2 (2.3) | 4 (4.7) |  |
| **TR score** |  |  |  |
| Whole patient group (n=86) | 1.7 ± 0.8 | 1.8 ± 0.9 | 0.145 |
| Longest lead age <10 years (n=46) | 1.6 ± 0.7 | 1.6 ± 0.8 | 1.000 |
| Longest lead age ≥10 years (n=40) | 1.8 ± 0.7 | 2.0 ± 0.8 | 0.058 |

*, P-values for Chi-square (TR severity) and paired t-test (TR score change)

Abbreviations: TLE, transvenous lead extraction; TR, tricuspid regurgitation

**Supplementary Table S3.** Details on TR aggravation or improvement following TLE.

|  | **Pre-TLE** | **Post-TLE** | **Longest lead age** |
| --- | --- | --- | --- |
| **Aggravation in TR** (n=6) |  |  |  |
| n=1 | Minimal | Severe | 10.1 years |
| n=2 | Mild | Severe | 9.0 and 21.6 years |
| n=1 | Minimal | Moderate | 9.4 years |
| n=2 | Mild | Moderate | 10.3 and 11.6 years |
| **Improvement in TR** (n=5) |  |  |  |
| n=1 | Severe | Moderate | 6.8 years |
| n=1 | Moderate | Mild | 27.0 years |
| n=3 | Mild | Trivial | 1.0, 7.4, and 8.3 years |

Significant TR aggravation (defined as an increase of at least 1 grade with a post-TLE TR ≥ moderate)

was identified in 6 of 86 (7.0%) patients whereas TR improvement (defined as a decrease of at least 1 grade)

was observed in 5 of 86 (5.8%) patients following TLE.

Abbreviations: TLE, transvenous lead extraction; TR, tricuspid regurgitation
